# Removal of Intra-Thoracic Projectiles

**Published:** 1918-12

**Authors:** 


### *Removal of Intra-thoracic Projectiles.* By Dr. ROBERT DIDIER. *Bulletin de la Société de Chirurgie de Paris,* April 30, 1918.

The author has done 25 operations for the removal of intra-thoracic projectiles. One of them was not finished, because the shell-fragment was found close against the inferior vena cava.

Among the 25 cases, 15 removals were effected simply by means of forceps, under X-ray control, without opening the chest. The projectiles were extracted five times from the lungs, once from the pericardium, thrice from the diaphragm, five times from the pleura, and once from the liver.

In the ten other cases, the removal was attempted only after wide opening of the chest. The projectiles were found five times in the hilum, twice in the lungs, thrice in the mediastinum.

Two deaths ensued from these operations.

The author considers that the removal of intra-thoracic projectiles should only be attempted for fragments presenting a certain size, and occasioning haemoptysis or abscesses.

